# Genetics and pathophysiology of diffuse idiopathic skeletal hyperostosis

**DOI:** 10.3389/fendo.2026.1745930

**Published:** 2026-02-17

**Authors:** Wenhao Ji, Wanlei Yang, Shikang Su, Simin Sun, Huanhao Cai, Kangnan Wang, Bin Pan, Yu Qian

**Affiliations:** The First Affiliated Hospital of Zhejiang Chinese Medical University (Zhejiang Provincial Hospital of Chinese Medicine), Hangzhou, Zhejiang, China

**Keywords:** diagnosis, diffuse idiopathic skeletal hyperostosis, genetics, ossification, pathophysiology

## Abstract

Diffuse Idiopathic Skeletal Hyperostosis (DISH) is a systemic condition primarily characterized by flowing ossification along the anterolateral aspects of the spine, often leading to back pain, dysphagia, and an increased risk of spinal fractures. Despite its significant clinical burden and high prevalence among the elderly, DISH remains underdiagnosed and poorly understood, with no disease-modifying therapies currently available. This article provides a comprehensive review of the genetic variations and environmental factors involved in the pathogenesis of DISH, encompassing recent research progress in inflammation, metabolism, pathogenic genes, and animal models. It also critically highlights the current challenges and future directions in DISH research.

## 1 Introduction

**Figure 1:**
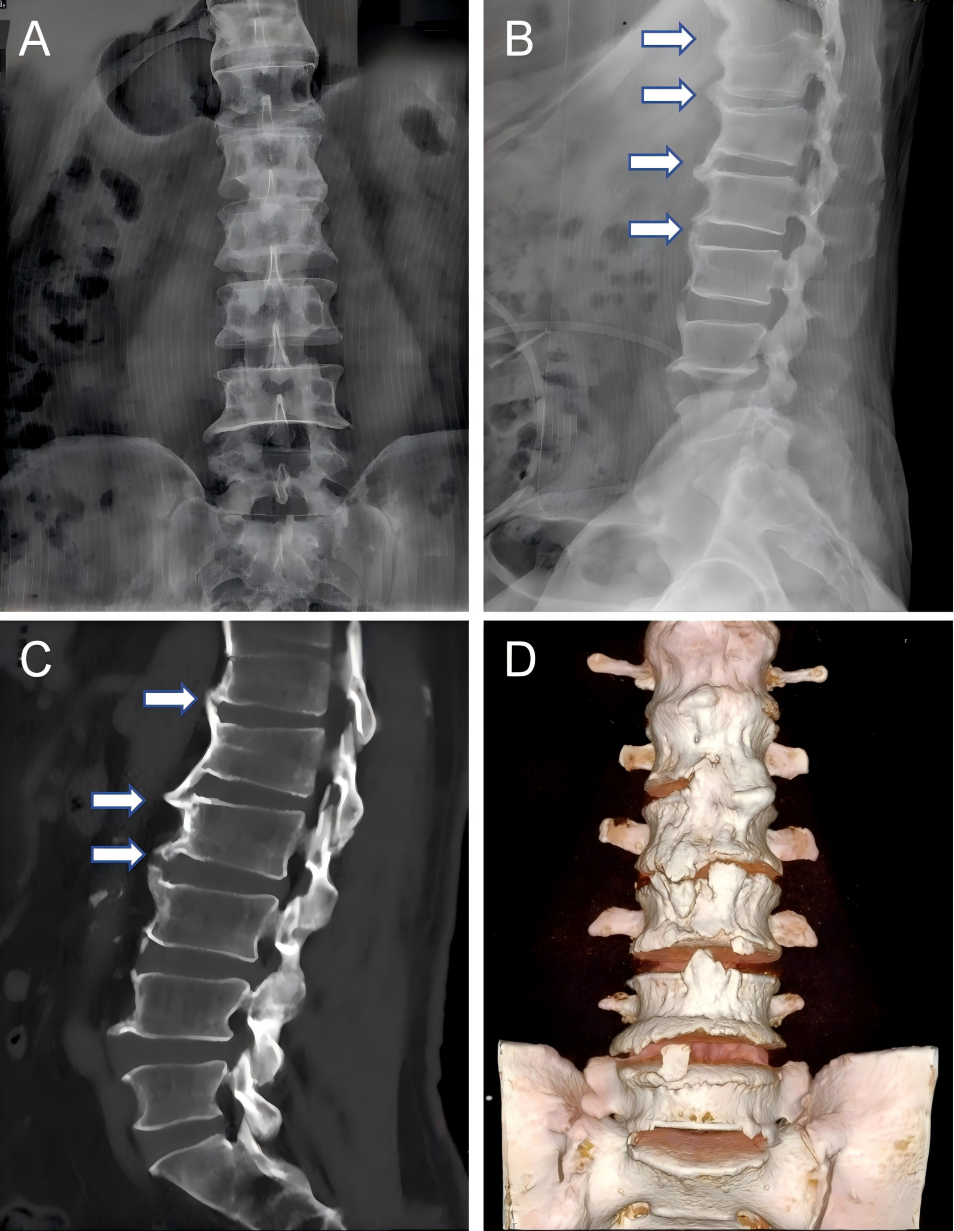
Representative imaging features of DISH in an elderly male patient. Flowing, bridging ossification is present anterior to the vertebral bodies (arrows). The sacroiliac joints show no significant erosion, sclerosis, or bony ankylosis. **(A)** Anteroposterior radiograph of the lumbar spine. **(B)** Lateral radiograph. **(C)** Sagittal CT reconstruction. **(D)** Three-dimensional CT reconstruction.

**Figure 2:**
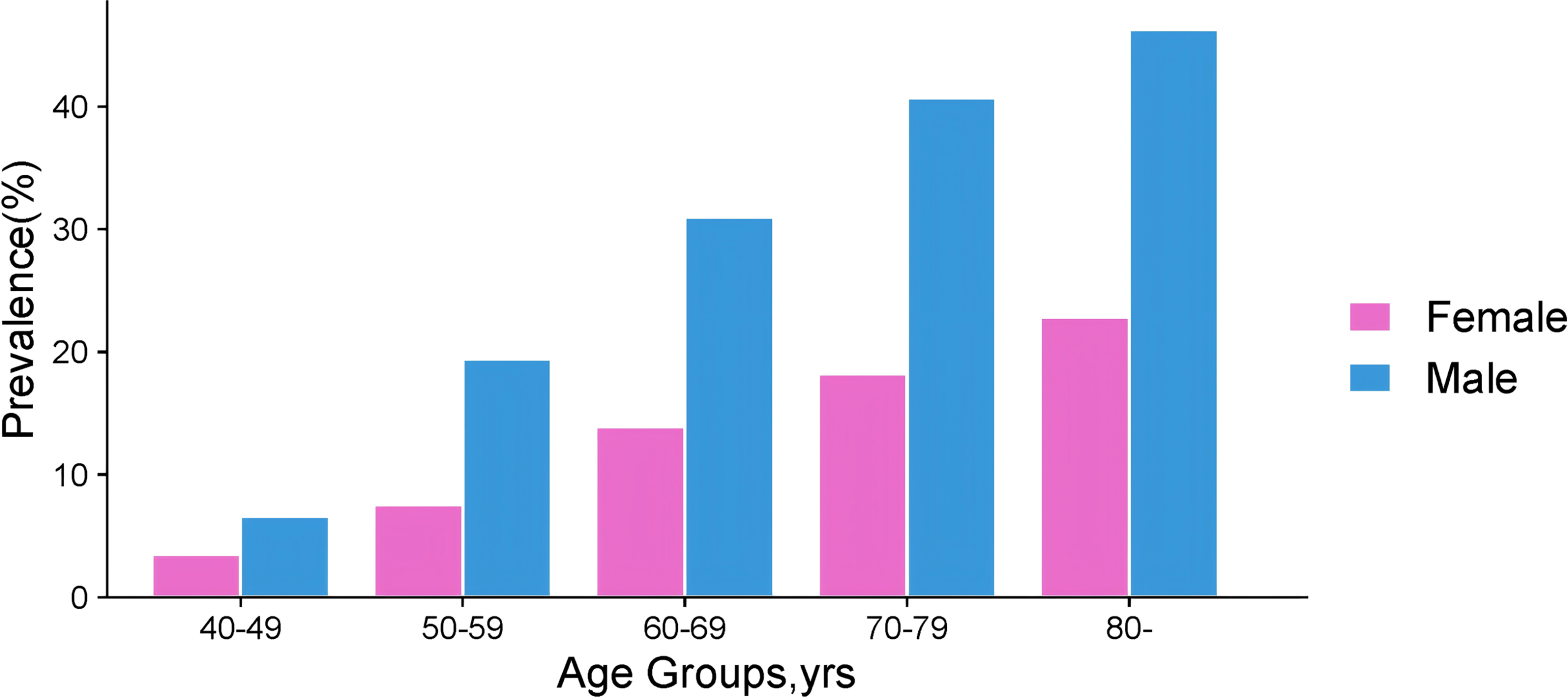
Prevalence of DISH across studies (4, 111–113).

Among musculoskeletal disorders, diffuse idiopathic skeletal hyperostosis (DISH) ranks as the second most prevalent form of arthritis, following osteoarthritis (OA) (1, 2). DISH is characterized by persistent ossification of tendon and ligament attachment points, especially in the axial bones dominated by the thoracic vertebrae, but the surrounding joints may also be involved (3, 4). (The characteristic radiographic appearance of DISH is exemplified in **Figure 1**). Globally, the prevalence of DISH varies significantly, ranging from 2.9% to 44.0%, influenced by factors such as the studied population, diagnostic criteria, and age distribution (4–8). In addition, **Figure 2** shows the prevalence data of DISH in different studies, indicating that the incidence rate of DISH varies by gender, with the proportion of men and women about 2:1, and the incidence rate increases with age (9, 10).

Since its initial description by Forestier and Rotes Querol in 1950, the diagnostic criteria for DISH have undergone a process from early initial description to gradual refinement and emphasis on early diagnosis (11). The radiological criteria proposed by Resnick and Niwayama in 1976 have been widely cited in the literature and are one of the main bases for the current diagnosis of DISH (12). However, there is still a lack of consensus on whether and how to include the criteria for disc height and peripheral manifestations (11)(**Table 1** summarizes the main diagnostic criteria from 1950 to the present, including imaging methods and diagnostic points). In addition, because the clinical symptoms of DISH have historically been considered mild and rarely require drug treatment, it has always been regarded as an entity of little clinical significance and received less attention from researchers (13). As a result, its pathophysiological mechanisms remain unclear, and it is often regarded as a variant of OA (14). Consequently, despite its high prevalence, health professionals are frequently unfamiliar with DISH, leading to it being overlooked or misdiagnosed (1). In fact, although some DISH patients may be asymptomatic, a significant number of DISH patients experience a variety of clinical symptoms, such as spinal pain and stiffness, increased risk of fractures, airway obstruction, or nerve tissue damage, that severely affect quality of life and may even lead to disability (15–18).

**Table 1:** Evolution of diagnostic criteria for DISH.

Year	Source/Author	Imaging modality	Criteria highlights (spine)	Criteria highlights (Peripheral involvement)
1950	Forestier et al. (114)	X ray	Continuous or discontinuous, candle wax-like bone hyperplasia is observed anterior to the vertebral bodies and intervertebral discs, appearing distinct from the anterior longitudinal ligament.	There are no pathological changes in the sacroiliac joint, but thickening and increased density of the iliac crest edge may lead to false joint fusion.
1971	Julkunen et al. (48)	X ray	At least two contiguous, well-defined bony bridges are present between the vertebrae of the thoracic spine.	Not mentioned.
1974	Harris et al. (115)	X ray	The dorsal spine should have hypertrophic bone spurs, mainly located on the right side, and at least two bone bridges should be present.	No sacroiliitis.
1974	Vernon-Roberts et al. (116)	X ray	syndesmophytes, characterized by bony flanges or bridges, typically located on the right anterolateral aspect of the vertebral bodies across two or more contiguous vertebrae.	Not mentioned.
1976	Resnick and Niwayama (12).	X ray	1.There is at least 4 consecutive running water ossification at the anterolateral margin of adjacent vertebrae, and osteophytes form at the intervertebral disc junction; 2.There was no obvious intervertebral disc degeneration, and the vertebral space height was normal; 3. Absence of facet joint anakylosis.	Absence of sacroiliac inflammatory changes.
1985	Utsinger (117)	X ray	Definite DISH: Flowing ossification spanning four or more contiguous vertebral bodies.	Possible DISH: Flow ossification of two consecutive vertebral bodies accompanied by symmetrical peripheral bone hyperplasia.
2001	Rogers and Waldron (118)	Not applicable	Hyperostosis of the spine characterized by involvement of at least three contiguous vertebrae, with or without accompanying ankylosis.	Calcification or ossification affecting spinal ligaments and/or entheses, including extra-spinal locations.
2017	Oudkerk et al. (119)	CT	Improved Resnick criteria: 1. At least four consecutive vertebral bodies or three consecutive disc levels have bridging ossification. 2. Osteophyte and vertebral body should be formed at an Angle greater than 90°. 3. It may be accompanied by mild or moderate degenerative disc changes	Not mentioned.
2019	Kuperus et al. (120)	CT	Early-Phase DISH: A complete bone bridge is present in one spinal segment, while the neighboring segment shows a nearly complete bridge, and the next adjacent segment exhibits early bone formation. Alternatively, three consecutive segments may display nearly complete bridging.	Not mentioned.

Despite the high prevalence of DISH and its potentially severe clinical impact, there are currently no disease-modifying therapies available to halt or reverse the pathological ossification process. Current management is primarily palliative, focusing on symptom relief and addressing complications (19). Consequently, in-depth exploration of the genetic and pathophysiological mechanisms of DISH will help reveal its pathogenesis and provide a theoretical foundation for the development of effective treatments.

**Figure 3:**
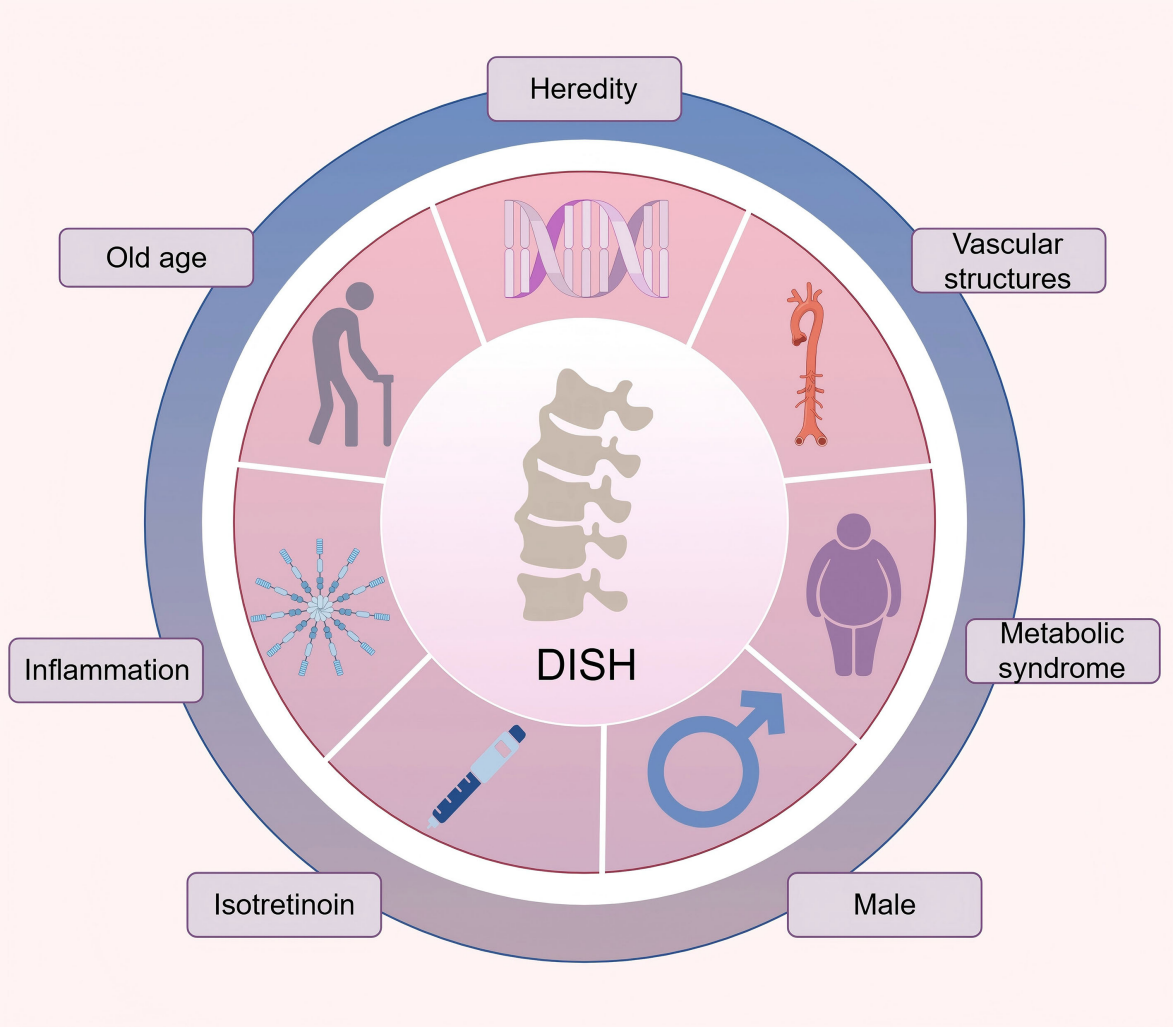
Pathological factors associated with DISH.

In the past few decades, clinical and epidemiological studies have demonstrated that DISH arises from a multifactorial etiology involving genetic predisposition, gender, age, metabolic influences, and so on, highlighting the complex interplay between genetic and environmental contributors (9, 19, 20). (**Figure 3**). In recent years, with the application of gene sequencing technology, genome-wide association studies (GWAS), immunohistochemistry and other technologies, it has provided a powerful means for discovering gene check points and pathophysiological mechanisms related to DISH, and the research on the pathogenesis of DISH has made some progress. This review synthesizes the latest knowledge regarding the genetics and pathophysiology of DISH, critically analyzes the obstacles in current research, with the aim of enhancing the comprehensive understanding of this disease and providing valuable references for scientific research and clinical practice.

## 2 Pathophysiology

### 2.1 DISH ossification-related signaling pathway

DISH ossification is a complex process. It is a heterotopic ossification (HO) of ligaments and tendons under the combined action of growth factors, molecular signals and cytodynamics.

#### 2.1.1 Mesenchymal stem cell

The abnormal differentiation of mesenchymal stem cell (MSC) in specific anatomical locations is a critical mechanism driving new bone formation in DISH (21). MSCs are primarily located in the outer fibrocartilage of entheseal sites, bony eminences at vertebral margins, and sesamoid bones, with the potential to proliferate and differentiate into osteoblasts or chondrocytes (21–23). Under the influence of metabolism-related growth factors, MSCs are activated. Insulin-like growth factors (IGF-1/2), through signaling with the IGF-1 receptor, induce osteogenic differentiation of these stem cells and enhance their osteogenic potential (24, 25). Bone morphogenetic proteins (BMPs) induce the differentiation of MSCs into chondrocytes, form new bone through endochondral ossification, and participate in the mineralization of fibrocartilage at the tendon attachment site and the growth of new bone (26, 27). Transforming growth factor-beta (TGF-β), in coordination with mechanical stress, influences the differentiation of mesenchymal cells into fibrocartilage or bone tissue (26, 27). Additionally, the function of MSCs derived from spinal ligaments is influenced by epigenetic processes; for example, demethylation of the *WNT5A* and *GDNF* genes enhances their osteogenic activity, suggesting that epigenetic modifications promote abnormal ossification by releasing transcriptional inhibition (28). This suggests that DNA methyltransferase inhibitors may be a potential treatment method, although their specificity for spinal entheses has not been confirmed.

#### 2.1.2 Wnt/β-catenin signaling pathway

The Wnt/β-catenin signaling pathway is a highly conserved and crucial pathway in skeletal development and homeostasis. Its aberrant activation is a significant mechanism driving pathological heterotopic bone formation in DISH (29, 30).

Clinical studies have found that serum levels of the Wnt pathway antagonist Dickkopf-1 (DKK1) are significantly lower in patients with DISH, suggesting systemic activation of Wnt signaling *in vivo (*31). Interestingly, another potent Wnt antagonist, sclerostin (SOST), has been reported to be elevated in the serum of DISH patients (32). This may represent a compensatory mechanism in response to the pathological ossification in DISH, although the specific underlying process remains unclear.

Transgenic mouse models powerfully demonstrate the pathogenic role of Wnt/β-catenin overactivation in DISH. Conditional activation of β-catenin in chondrocytes and intervertebral disc cells of *β-catenin(ex3)^Col2CreER^* and *β-catenin(ex3)^Agc1CreER^* mice successfully recapitulates the characteristic spinal phenotypes of human DISH, including extensive osteophyte formation and vertebral fusion (30).

Furthermore, recent studies have identified the lectin Galectin-3 as a key upstream factor activating the Wnt/β-catenin pathway in DISH (33). Bone marrow-derived mesenchymal stem cells (BMSCs) from DISH patients exhibit a high-secretory phenotype for Galectin-3, and its expression level directly correlates with the abnormally enhanced osteogenic differentiation capacity of these cells. Galectin-3 activates downstream signaling by directly binding to β-catenin and promoting its nuclear translocation, whereas inhibition of the Wnt/β-catenin pathway blocks the pro-osteogenic effects of Galectin-3. Upon pathway activation, the canonical β-catenin/TCF transcriptional program drives the expression of osteogenic genes, promoting BMSC osteogenic differentiation (33). Concurrently, it upregulates key degradative enzymes such as matrix metalloproteinase 13 (MMP13) and aggrecanases (ADAMTS4/5), which disrupt the cartilage matrix and create a permissive environment for heterotopic osteophyte deposition. Experimentally, knocking out the *Mmp13* or *Adamts5* gene in mice with activated β-catenin significantly alleviates the phenotypes of intervertebral disc destruction and osteophyte formation (30).

In summary, overactivation of the Wnt/β-catenin pathway constitutes a crucial signaling axis driving pathological ossification in DISH. This understanding provides potential theoretical targets for future development of disease-modifying therapies aimed at various components of this pathway.

#### 2.1.3 Nuclear factor κB signaling pathway

Nuclear factor κB (NFκB) plays a regulatory role in the expression of multiple genes involved in cell growth and division as well as in the differentiation of multipotent cells (20). In ligament mesenchymal cells, NFκB activation is linked to osteoblastic differentiation. Kosaka et al. obtained tissue samples from patients with ossification of the spinal ligaments (OSL)/DISH and from individuals with non-ossified spinal ligaments during surgery, and found that the number of NFκB-positive samples was significantly higher in the OSL/DISH group, along with elevated levels of PDGF-BB and TGF-β1 in ligament tissues (34). The authors proposed that these growth factors may activate the NFκB signaling pathway, promoting the nuclear translocation of NFκB, which in turn regulates the expression of related genes (such as Sonic Hedgehog, Twist, and BMP-4) and thereby influences the differentiation of multipotent cells. Furthermore, increased alkaline phosphatase (ALP) activity was observed in ligament cells from the OSL/DISH group, indicating their characteristics as osteoprogenitor cells. The study suggests that NFκB activation may play an important role in the pathogenesis of DISH. Notably, this pathway was more significantly activated in patients complicated with non-insulin-dependent diabetes mellitus (NIDDM), highlighting a potential mechanism linking metabolic disturbances to enhanced local ossification signaling (34).

#### 2.1.4 Bone morphogenetic protein 2 signaling pathway

Bone morphogenetic protein (BMP) 2 signaling is one of the key drivers in the ossification process of DISH (20). It activates the Smad1/5/8 signaling pathway, enhancing the differentiation of MSCs into osteoblasts (35). Histological investigations have revealed a distinct spatial distribution of BMP−2, TGF−β, and decorin within ossified lesions from patients with DISH (36). Specifically, BMP−2 expression is markedly elevated in woven bone and in muscle tissue adjacent to the ossification sites, suggesting its pivotal role in the initiation of ectopic bone formation and the osteogenic transformation of muscle−derived cells. Concurrently, the enrichment of TGF−β and decorin in the bone matrix points to their cooperative involvement in regulating extracellular matrix assembly and mineralization. These findings provide direct evidence for the molecular pathological basis underlying the heightened susceptibility to severe ectopic ossification in individuals with DISH (36–38). Critically, functional characterization of the heterozygous *ALK2* p.K400E mutation identified in DISH patients demonstrates enhanced signaling to osteogenic BMPs, providing a direct genetic-mechanistic link (39). The convergence of histological and genetic evidence strongly positions hyperactive BMP signaling as a key driver in DISH, although its precise interaction with other pathways in disease initiation needs clarification.

### 2.2 Metabolic factors

Metabolic disorders are one of the important pathogenic factors of DISH, which not only contribute to the occurrence of the disease but also are related to its phenotypic heterogeneity and progression rate. The interplay between visceral obesity, insulin resistance, and related endocrine pathways creates a permissive environment for ectopic bone formation at ligamentous and entheseal sites.

#### 2.2.1 Obesity

Obesity, particularly visceral adiposity, is one of the most robust clinical associations with DISH. The prevalence of DISH reaches 18% in patients aged 50 years or younger who suffer from severe obesity, a figure substantially higher than that in the general population (40). This means that the metabolic environment of severe obesity can strongly accelerate the ossification process much earlier than the traditional onset age of DISH. In obesity, visceral adipose tissue (VAT) secretes various adipokines, such as leptin and adiponectin (41). Especially the increase in leptin level has been confirmed in female patients with ossification of spinal ligament (42). Leptin promotes osteogenesis through the JAK/STAT signaling pathway, stimulates stromal cells to differentiate into osteoblasts, increases osteoblast proliferation, and inhibits osteoclast production (43), thereby promoting local ossification progression. Moreover, the visceral adiposity index (VAI), a substitute marker of visceral fat dysfunction, is significantly elevated in patients with the “fast ossifier” phenotype of DISH (44). This association is particularly pronounced in women, indicating that visceral obesity serves as a critical driver of accelerated ossification and concomitant early trabecular deterioration in this subgroup. These findings underscore that weight management and metabolic control may represent potential strategies to mitigate disease progression.

#### 2.2.2 Type 2 diabetes mellitus

There is a close connection between DISH and type 2 diabetes mellitus (T2DM), with the prevalence of DISH in T2DM patients reaching 13% (4, 45–47). Even in cases where obesity is controlled, patients with impaired glucose metabolism show a higher incidence of DISH (48). Patients with type 2 diabetes often experience hyperinsulinemia, which promotes osteoblast activity and bone formation. Insulin may produce similar anabolic effects as IGF-1 through homology in its molecular structure and stimulate osteoblast differentiation (49). In addition, in the state of diabetes, the level of SOST in DISH patients is reduced (46), which weakens the inhibition of Wnt signaling pathway, and then promotes the expression of osteogenesis related genes. It is worth noting that DISH has not been recorded in patients with T1DM (50).

#### 2.2.3 Growth hormone

Growth hormone (GH) represents another significant metabolic factor influencing the development of DISH. GH can directly act on osteoblasts to promote their proliferation and differentiation, while also stimulating the production of IGF-1, which enhances the proliferation of bone and cartilage cells (51, 52). A case-control study observed that serum GH levels were significantly lower in asymptomatic DISH patients compared to symptomatic ones, thereby proposing serum GH as a potential biomarker for monitoring clinical inflammatory activity (53). However, this study had notable limitations: a small sample size, a cross-sectional comparison design, and a failure to fully account for confounding effects from symptomatic treatments on GH levels. Consequently, the precise role of the GH/IGF-I axis in osteophyte formation in DISH, remains to be elucidated through more rigorous, large-scale longitudinal studies.

### 2.3 Inflammation

DISH was traditionally regarded as a non-inflammatory disease, but recent evidence indicates that inflammation significantly contributes to its pathogenesis, especially in the early stages (41, 54). Multiple studies have demonstrated the presence of inflammatory markers, such as elevated C-reactive protein (CRP), alkaline phosphatase (ALP), and interleukin-17 (IL-17), in patients with DISH (55, 56). Imaging studies further strengthen this connection, as DISH patients frequently exhibit MRI findings such as bone marrow edema (BME) and fatty deposits at vertebral corners—features commonly observed in inflammatory conditions like axial spondyloarthritis (SpA) (57, 58). This overlap suggests shared inflammatory mechanisms underlying bone proliferation in both diseases. In addition, ultrasound studies on the peripheral tendon ends of DISH patients have found a high incidence of tendinopathy, with a relatively high incidence of erosion in some areas and increased Power Doppler (PD) activity in some areas (59). This highly suggests the presence of local inflammation, and it is speculated that inflammatory changes may occur earlier than structural changes in new bone formation in the early stages of the disease (60).

This was further confirmed by micro-X-ray computed tomography scanning analysis, which revealed widespread destruction and remodeling of the original cortex at the anterior vertebral margins in DISH patients, accompanied by the formation of trabecular bone structure within the new bone (54). This active process of bone erosion and reconstruction suggests the presence of a localized inflammatory process, rather than mere bone hyperplasia.

### 2.4 Vascular factors

The influence of vascular factors on DISH is multifaceted, involving direct vascular contributions to bone growth as well as a modulating effect on the distribution and progression of ossification (61).

Specifically, angiogenesis is crucial for osteoblast proliferation and a key link in skeletal development and bone repair (62). Vascular endothelial cells guide osteoclast and osteoblast precursors to specific areas through various mediators such as vascular endothelial growth factor (VEGF) and basic fibroblast growth factor (bFGF). Meanwhile, bone endothelial cells can respond to bone regulators such as estrogen and PTH (62). This active process of HO involving specific endothelial differentiation and subsequent bone cell migration can partially explain the new bone formation at entheseal sites in patients with DISH. In addition, radiological studies have revealed an increase in the number and width of nutrient foramina within the vertebrae of DISH patients, indicating a heightened demand for blood supply in the ossifying regions and underscoring the intimate link between hypervascularity and active bone growth (63).

The morphology and distribution of osteophytes in DISH are closely associated with positional changes of the aorta. From the T5 to L4 spinal levels, regardless of whether the aorta is situated on the left or right side, most osteophytes form opposite to it, while those in close proximity to the aorta tend to appear flat or concave (64, 65). This suggests that the pulsatile movement of the aorta inhibits ossification on the ipsilateral side by providing mechanical displacement that prevents stable bone growth.

### 2.5 Isotretinoin

A series of case reports and small retrospective series have raised the hypothesis of an association between isotretinoin use and the development of DISH (66–69). For instance, in an uncontrolled study of 96 cystic acne patients treated with isotretinoin, 26% exhibited progressive development of small bone spurs over a 2-year period (70). However, the absence of a control group and the inherent limitations of retrospective case series preclude any definitive conclusions regarding causality. Currently, this association remains a preliminary observation that warrants investigation in controlled, prospective epidemiological studies.

## 3 Genetics

### 3.1 Familial cases and hereditary patterns in DISH

Differences in the prevalence of DISH between different countries and ethnic groups suggest that genetic factors may play a role in its pathogenesis. Several studies have reported familial and population aggregation of DISH, further indicating that the occurrence of DISH is related to genetic factors (71–75). According to Bruges-Armas et al., the transmission pattern of DISH is consistent with autosomal dominant monogenic diseases. There is no skipped-generation inheritance. And approximately 50% of the offspring of affected parents are also affected, regardless of whether the offspring are male or female (73).

However, the quest to identify a specific genetic locus has been complex. An early study by Gorman et al., which performed tissue typing on two affected siblings, found that they only shared alleles of DRB3 and C sites, which are also common in the general population. Therefore, their research suggests that if DISH contains a genetic component, it may not be related to human leukocyte antigen(*HLA*) status (71).

### 3.2 Specific genetic mutations linked to DISH

So far, a large number of genetic studies targeting DISH patients have investigated the susceptibility genes of the disease through methods such as whole exome sequencing and GWAS, revealing the genetic risk factors for DISH occurrence (76, 77) (**Table 2** shows the genes and genetic variant information related to DISH). We have reviewed some important genes related to bone and cartilage formation in candidate gene association studies below.

**Table 2:** Genes associated with DISH (gene function source: genecards database).

Gene	SNVs	Gene function	Study design	Country	Time	Ref
*COL6A6*	rs200963433	Collagen VI functions as a cell-binding protein.	CGAS	America	2023	Thomas M et al. (81)
*TGF-β1*	rs2241716	Plays a critical role in bone remodeling by acting as a potent stimulator of osteoblastic bone formation, promoting chemotaxis, proliferation, and differentiation in committed osteoblasts.	CGAS	America	2023	Thomas M et al. (81)
*TLR1*	rs145135062	Engages in the innate immune defense against microbial pathogens by activating MYD88 and TRAF6 signaling pathways, resulting in NF-kappa-B activation, cytokine release, and the initiation of inflammatory processes.	CGAS	America	2023	Thomas M et al. (81)
*RUNX2*	NA	This gene encodes a transcription factor essential for osteoblast differentiation and skeletal development. It plays a pivotal role in osteoblast maturation and is critical for both intramembranous and endochondral bone formation.	GWAS	Britain	2023	Sethi et al. (1)
*IL11*	rs4252548	This gene promotes the proliferation of hematopoietic stem cells and megakaryocyte progenitor cells, while also inducing megakaryocyte maturation as a cytokine, ultimately enhancing platelet production.	GWAS	Britain	2023	Sethi et al. (1)
*GDF5*	NA	This gene encodes a growth factor that plays a key role in bone and cartilage formation.	GWAS	Britain	2023	Sethi et al. (1)
*CCDC91*	NA	This gene plays a role in facilitating transport processes from the Golgi apparatus to lysosomes.	GWAS	Britain	2023	Sethi et al. (1)
*NOG*	NA	This gene acts as an inhibitor of BMP signaling, suppressing chondrocyte differentiation through interactions with GDF5 and GDF6.	GWAS	Britain	2023	Sethi et al. (1)
*ROR2*	NA	This gene encodes a tyrosine protein kinase receptor that may play a role in the early stages of chondrocyte formation.	GWAS	Britain	2023	Sethi et al. (1)
*SLC30A8*	NA	The protein encoded by this gene functions as a zinc efflux transporter, facilitating zinc accumulation in intracellular vesicles and playing a regulatory role in insulin secretion.	GWAS	Britain	2023	Sethi et al. (1)
*PIK3R1*	NA	This gene is crucial for insulin metabolism, and mutations in this gene are linked to insulin resistance.	GWAS	Britain	2023	Sethi et al. (1)
*CHRDL2*	NA	This gene may suppress BMP activity by preventing BMP-receptor interactions, exerting a negative regulatory effect on cartilage formation and regeneration in immature mesenchymal cells.	GWAS	Britain	2023	Sethi et al. (1)
*SUPT3H*	NA	This gene may be a transcription activator.	GWAS	Britain	2023	Sethi et al. (1)
*UQCC1*	NA	This gene is essential for the assembly of the ubiquinol-cytochrome c reductase complex and plays a role in the translation and stability of cytochrome b.	GWAS	Britain	2023	Sethi et al. (1)
*ANKFN1*	NA	This gene may be involved in regulating neuronal function.	GWAS	Britain	2023	Sethi et al. (1)
*HAO1*	NA	This gene is predominantly expressed in the liver and pancreas, and its encoded protein exhibits the highest activity on glycolate.	GWAS	Britain	2023	Sethi et al. (1)
*TMX4*	NA	This gene encodes a protein belonging to the disulfide isomerase family, which is localized in the endoplasmic reticulum and facilitates protein folding and thiol-disulfide exchange reactions.	GWAS	Britain	2023	Sethi et al. (1)
*PLCB1*	NA	This gene encodes an enzyme that catalyzes the hydrolysis of phosphatidylinositol 4,5-bisphosphate (PIP2) into diacylglycerol (DAG) and inositol 1,4,5-trisphosphate (IP3), playing a key role in intracellular signaling pathways activated by G protein-coupled receptors.	GWAS	Britain	2023	Sethi et al. (1)
*POLD3*	NA	This gene encodes the 66 kDa subunit of DNA polymerase delta, a key enzyme involved in DNA replication and repair processes.	GWAS	Britain	2023	Sethi et al. (1)
*ENPP1*	Heterozygous missense variation	This gene regulates pyrophosphate levels, playing a critical role in bone mineralization and soft tissue calcification.	CGAS	Japan	2022	Hajime et al. (87)
*ALK2*	Heterozygous missense variation	NA	CGAS	NA	2020	Sho Tsukamoto et al. (39)
*PPP2R2D*	rs34473884	The PPP2R2D protein is a serine/threonine protein phosphatase that regulates fundamental cellular processes by dephosphorylating target substrates.	WES	Portugal	2020	Parreira B et al. (99)
*ACVR1*	Heterozygous missense variation	This gene encodes a type I receptor for BMP and plays a role in diverse biological processes, such as the development and regulation of bone, heart, cartilage, nervous, and reproductive systems.	CGAS	NA	2019	Aditi et al. (92)
*RSPO4*	rs146447064/rs149154047	The encoded protein may play a role in activating Wnt/β-catenin signaling pathways.	WGWLA and IBD/IBS	Portugal	2017	Ana Rita Couto et al. (97)
*LEMD3*	rs201930700	This gene functions as a specific inhibitor of TGF-β, activin, and BMP signaling pathways through its interaction with R-SMAD proteins.	WGWLA and IBD/IBS	Portugal	2017	Ana Rita Couto et al. (97)
*FGF2*	rs1476217/ rs3747676	This gene plays a critical role in regulating cell survival, division, differentiation, and migration.	CGAS	Korean	2012	Jun et al. (84)
*COL6A1*	rs2236486	Collagen VI is a key structural component of microfibrils, and mutations in this gene are associated with Bethlem myopathy. Mutations in the genes encoding collagen VI subunits lead to Bethlem myopathy, an autosomal dominant disorder.	CGAS	Japan	2005	Tsukahara et al. (80)

SNVs, Single-Nucleotide Variants; WGWLA, whole-genome-wide linkage analysis; WES, whole exome sequencing; GWAS, Genome - Wide Association Studies; CGAS, Candidate gene association study.

#### 3.2.1 COL6A1and COL6A6

Both *COL6A6* and *COL6A1* belong to the collagen gene family, which forms a critical component of the extracellular matrix (ECM) and is involved in membrane or intracondral osteogenesis (78, 79). Genetic studies have linked single nucleotide polymorphisms (SNPs) in *COL6A1* and *COL6A6* to DISH (80, 81). Specifically, the association of *COL6A1* with DISH was significant in a Japanese cohort but not observed in a Czech population, suggesting the existence of population-specific genetic effects (80). These variants may contribute to the pathogenesis of DISH by altering the structure of the ECM, thereby creating a scaffold that facilitates HO, although the precise underlying mechanisms require further clarification.

#### 3.2.2 FGF2

Fibroblast growth factor 2 (*FGF2*) plays a multifaceted role in bone metabolism, promoting the recruitment of BMSCs and stimulating bone formation (82, 83). Jun et al. found through sequencing that two specific SNPs within the *FGF2* gene (rs1476217 and rs3747676) that are significantly associated with DISH (84). This finding suggests that dysregulated *FGF2* signaling may disrupt normal bone remodeling processes, thereby contributing to the pathological HO seen in DISH.

#### 3.2.3 ENPP1

The Ectonucleotide pyrophosphatase/phosphodiesterase 1 (*ENPP1*) gene encodes a transmembrane enzyme that generates inorganic pyrophosphate (PPi), a potent inhibitor of ectopic mineralization, by hydrolyzing high-energy phosphate bonds (85, 86). An identified heterozygous missense mutation (c.1352A>G, p.Y451C) within the catalytic domain of *ENPP1* has been linked to DISH (87). This mutation leads to decreased PPi levels in patients, consequently weakening the inhibition of hydroxyapatite crystal growth. This manifests clinically as a spectrum of ectopic calcification symptoms in patients, including ossification of paraspinal ligaments and calcification of the Achilles tendon (87, 88).

However, a critical paradox has emerged from recent animal studies. A knock-in mouse model carrying the corresponding *ENPP1* Y433C mutation (corresponding to human Y451C mutation) showed no significant bone microstructural abnormalities or ectopic calcification at various time points (89).

This discrepancy may arise from compensatory effects of proteins like *ENPP3* in mice, while DISH pathogenesis involves polygenic interactions and environmental factors (89). Species differences may also contribute to the discrepancy between the model and clinical phenotypes. Resolving this paradox is crucial for fully understanding the role of *ENPP1* in DISH and requires further collection of human cases and the development of more precise animal models.

#### 3.2.4 ACVR1/ALK2

The Activin A receptor type I (*ACVR1*) gene encodes the bone morphogenetic protein (BMP) type I receptor *ALK2*, a pivotal component of the BMP signaling pathway essential for bone formation (90, 91). In DISH, a specific heterozygous missense mutation (p.K400E) within the kinase domain of *ACVR1/ALK2* has been identified (92). This mutant receptor exhibits enhanced signaling in response to osteogenic BMPs but remains unresponsive to non-osteogenic ligands such as Activin A (39). This aberrant signaling leads to constitutive activation of the BMP-Smad pathway, resulting in the upregulation of osteogenic target genes like *ID1* and *Msx2 (*90, 92). Furthermore, BMP type II receptors can further enhance the BMP signaling activity mediated by p.K400E through phosphorylating it (39). Collectively, the p.K400E mutation provides direct genetic evidence that hyperactive BMP signaling is an important driver of pathological ossification in DISH.

#### 3.2.5 TGF-β1

*TGF-β1* is a pivotal cytokine in bone remodeling, which can promote the osteogenic differentiation of BMSCs while concurrently suppressing bone resorption by downregulating factors such as the osteoclast differentiation factor (93, 94). Evidence from both human studies and mouse models of HO has demonstrated activated TGF-β signaling at ectopic sites, and neutralization of TGF-β can alleviate HO progression (95). Genetically, an allelic analysis by Thomas M et al. revealed a significantly higher frequency of the SNP rs2241716 in the *TGF-β1* gene among DISH patients compared to global reference frequencies (81). This finding strongly supports *TGF-β1* as a susceptibility gene for DISH, implicating its enhanced signaling activity in the disease’s pathophysiology.

#### 3.2.6 RSPO4

R-spondin 4 (*RSPO4*), a member of the R-spondin family of secreted proteins, is a potent activator of the Wnt/β-catenin signaling pathway, which plays a critical role in cell proliferation and osteogenesis (96). A whole-genome-wide linkage and identity-by-descent (IBD)/identity-by-state (IBS) study conducted on families with coexisting DISH and chondrocalcinosis (CC) from the Azores islands identified the *RSPO4* gene as a potential candidate. Within this gene, nine genetic variants were discovered (73, 97). Notably, the rs146447064 variant was significantly more frequent in the control population, suggesting that it may confer a protective effect against the development of DISH/CC. This finding positions *RSPO4*-mediated Wnt signaling as a potentially important and modifiable pathway in the pathogenesis of DISH.

#### 3.2.7 PPP2R2D

*PPP2R2D* encodes a regulatory subunit of protein phosphatase 2A (*PP2A*), a serine/threonine phosphatase involved in diverse cellular processes (98). Whole exome sequencing of DISH patients from the Azores identified a specific variant (rs34473884) in *PPP2R2D* that showed a significant association with the DISH phenotype (99). While the precise molecular mechanism by which this variant contributes to HO remains unclear, its discovery supports the polygenic nature of DISH and suggests that dysregulation of protein phosphorylation may play a role in its pathogenesis.

### 3.3 GWAS

While candidate gene studies have provided valuable insights, GWAS offer a more comprehensive and unbiased approach to identifying genetic risk factors for DISH. A landmark GWAS identified ten significant genetic loci associated with the condition. These loci implicate a diverse set of genes with established roles in bone biology, including *RUNX2*, *IL11*, *GDF5*, and *NOG*, among others (1).

A key finding from this study was that the genetic variants associated with DISH were also significantly correlated with increased bone mineral density and content throughout the body. This suggests that DISH is not merely a localized spinal condition but may be a manifestation of a systemic predisposition to heightened bone formation. Furthermore, Mendelian randomization analysis provided robust evidence supporting a causal role for these identified genetic loci in the development of DISH (1).

These GWAS findings have precisely pinpointed the specific biological pathways driving DISH. Future research needs to validate these genes through *in vitro* experiments and animal models to elucidate their exact mechanistic roles in the pathological ossification process.

### 3.4 Epigenetics in DISH

While genetic factors provide important insights into DISH susceptibility, epigenetic mechanisms offer another layer of regulation that profoundly influences disease pathogenesis without altering the DNA sequence itself. Emerging evidence demonstrates that epigenetic modifications play crucial roles in regulating osteogenic differentiation in OSL.

DNA methylation represents a fundamental epigenetic mechanism governing gene expression in bone biology. In MSCs from patients with OSL, decreased DNA methylation in the promoter regions of the *WNT5A* and *GDNF* genes leads to their increased expression, which subsequently promotes osteogenic differentiation through the Wnt signaling pathway (28, 100).

Beyond DNA methylation, non-coding RNAs (ncRNAs) constitute another crucial epigenetic component in DISH pathogenesis. These RNA molecules, including microRNAs (miRNAs) and long non-coding RNAs (lncRNAs), fine-tune gene expression through post-transcriptional regulation and chromatin remodeling (101, 102). In the context of OSL, miR-181a-5p has been identified as significantly upregulated (103). This miRNA promotes osteogenic differentiation by targeting the transcription factor PBX1, which normally suppresses osteogenic genes. By reducing PBX1-mediated inhibition, miR-181a-5p facilitates the binding of pro-osteogenic transcription factors like *RUNX2* to promoter regions, thereby activating the osteogenic program (103).

### 3.5 Genetic evidence of DISH in animal models

In addition to the existence of genetic evidence of DISH in the human body, there is also a series of genetic evidence in the research of animal models.

A comprehensive radiological study of 2,041 skeletally mature dogs revealed an overall DISH prevalence of 3.8%, with incidence increasing with age and showing male predisposition (104). Among them, the prevalence rate of boxer dog was as high as 40.6%. The high incidence of DISH in this dog further supports that the occurrence of DISH is genetically related, and it can be used as an animal model to study DISH (105, 106). Notably, when dogs are used as animal models for spinal research, biomechanical problems need to be considered.

In addition, the *ENT1* knockout (*ENT1-/-*) mouse model has been extensively studied as a valuable tool for understanding DISH, as it replicates several key features of the disease. These include the progressive ectopic mineralization of spinal tissues and mineral deposits in non-spinal regions (107–109). This progressive calcification is thought to be caused by the loss of *ENT1*, which increases extracellular adenosine levels, thereby affecting pathways that regulate osteogenic differentiation and matrix mineralization (110).

In summary, studies on familial aggregation, candidate gene analysis, and GWAS have collectively established the significant role of genetic factors in the pathogenesis of DISH, revealing its complex polygenic background. However, it must be clearly recognized that most of these findings remain at the level of statistical associations, and their biological functions and specific mechanisms in disease development constitute the primary shortcoming in current understanding. Bridging this gap from association to mechanism will be a key focus of future research.

## 4 Future directions

When synthesizing the evidence for DISH pathogenesis, it is crucial to acknowledge the significant heterogeneity in the strength of available research. Some key discoveries cited in this review, such as specific etiological associations, certain serum biomarkers, and genetic risk loci, remain based on case reports, small-scale studies, or extrapolation from related disease models. The level of evidence for these findings is still preliminary or suggestive. Regarding the specific mechanisms of action of core signaling pathways in DISH, research directly derived from tissues of DISH patients or disease-specific models remains relatively limited. These evidence gaps and uncertainties precisely identify the priority areas that require focused efforts to address in future studies.

Despite recent advances in understanding the genetic and molecular mechanisms of DISH, translating these findings into clinical applications remains limited. Currently, there is no unified diagnostic standard for DISH. The most commonly used Resnick criteria strictly require ossification of the anterolateral aspects of four consecutive vertebrae, which overlooks early-stage and non-spinal manifestations of DISH. To improve early diagnosis and patient stratification, future efforts should develop standardized diagnostic criteria that integrate multimodal data. These may include imaging features of early, incompletely formed bony bridges, potential candidate molecular biomarkers, and possibly accompanying metabolic symptoms.

Currently, there are no disease-modifying therapies for DISH. Treatment is limited to symptom relief, with surgical intervention reserved for severe cases. Although key signaling pathways such as Wnt/β-catenin, BMP2-Smad, and NFκB are associated with the disease, targeted drugs that have entered clinical evaluation are lacking. Furthermore, while genetic studies have identified multiple risk loci and candidate genes, to date, apart from a few genes that have received preliminary functional characterization at the cellular and molecular level, the specific pathogenic mechanisms for the vast majority of reported genetic variants in DISH remain unknown. Moreover, the interaction between these genetic predispositions and the metabolic disturbances commonly seen in DISH patients (such as obesity and diabetes) has not been sufficiently explored. Therefore, future research must subject these statistical associations to rigorous functional validation to clarify how these risk alleles regulate target gene expression, affect protein function, and ultimately drive the pathological phenotype. This is not only an essential step for understanding DISH etiology but also the foundation for translating genetic discoveries into potential diagnostic markers and therapeutic targets.

## 5 Summary

DISH is a common systemic condition characterized by progressive HO at ligament and tendon attachment sites. The precise pathogenesis of DISH remains incompletely understood, though available evidence indicates that complex interactions among genetic predisposition, metabolic dysregulation, low-grade inflammation, and vascular factors collectively drive aberrant osteogenic differentiation of MSCs through dysregulated signaling pathways including Wnt/β-catenin, BMP-Smad, and NF-κB. Further investigation into the molecular mechanisms underlying ectopic bone formation in DISH is thus needed, along with identification of disease-associated genetic markers, to facilitate the development of early diagnostic tools and precision intervention strategies.
